# Transplant Trial Watch

**DOI:** 10.3389/ti.2022.10706

**Published:** 2022-08-01

**Authors:** Simon R. Knight

**Affiliations:** ^1^ Senior Clinical Research Fellow and Honorary Consultant Transplant Surgeon, Oxford Transplant Centre, Churchill Hospital, Oxford, United Kingdom; ^2^ Co-Director, Centre for Evidence in Transplantation, Nuffield Department of Surgical Sciences, University of Oxford, Oxford, United Kingdom

**Keywords:** liver transplantation, paediatric, systematic review, donor management, brain dead donors, randomised controlled trial

To keep the transplantation community informed about recently published level 1 evidence in organ transplantation ESOT and the Centre for Evidence in Transplantation have developed the Transplant Trial Watch. The Transplant Trial Watch is a monthly overview of 10 new randomised controlled trials (RCTs) and systematic reviews. This page of Transplant International offers commentaries on methodological issues and clinical implications on two articles of particular interest from the CET Transplant Trial Watch monthly selection. For all high quality evidence in solid organ transplantation, visit the Transplant Library: www.transplantlibrary.com.

**Systematic Review**

Liver Transplantation for Pediatric Hepatocellular Carcinoma: A Systematic Review.

*by Kakos, C. D., et al. Cancers (2022); 14(5): 02*.

## Aims

This study aimed to summarise all available evidence on the clinicopathological characteristics and oncological outcomes following liver transplantation (LT) among pediatric hepatocellular carcinoma (HCC) patients.

## Interventions

Electronic databases including MEDLINE, Scopus, Cochrane Library, and Web of Science were searched. Studies were screened and data were extracted by two independent reviewers.

## Participants

67 Studies were included in the review.

## Outcomes

The main outcomes were overall survival, disease-free survival and posttransplant complications.

## Follow-Up

5 years.

## CET Conclusion

This is a well-written report of a well-conducted systematic review in paediatric liver transplantation. Multiple databases were searched, and studies and data were extracted by two reviewers in duplicate. Sixty-seven studies reporting 245 patients in total were included from many different countries worldwide, published between 1985 and 2020. Each included study may have had only a few patients, range 1–25, with most studies reporting 1–2 patients only. The authors provide a general comment about the quality of included studies, and it would have been better to see individual studies formally quality assessed and possibly stratified for quality or by era of treatment. At mean follow up of 38.6 months, tumour recurrence was reported in 16.2% of patients, most commonly in the lungs and liver. 5-year disease free survival was 84.5%. At mean follow up of 46.8 months, overall survival was 84.8%, with tumour recurrence being the most common cause and this fits with the expected rate of tumour recurrence. 5-year overall survival was 74.3%. Liver transplantation to treat HCC in children offers long-term survival, and grafts from live donors showed a significant improvement compared to deceased donor grafts.

## Trial Registration

www.researchregistry.com (reviewregistry1310).

## Funding Source

No funding received.

**Randomised Controlled Trial**

Hemodynamic Effects of High-Dose Levothyroxine and Methylprednisolone in Brain-Dead Potential Organ Donors.

*by Van Bakel, A. B., et al. Transplantation (2022) [published ahead of print].*

## Aims

The aim of this study was to examine whether high-dose levothyroxine, high-dose methylprednisolone, or a combination of the two hormones, when administered early in the course of donor management, would lead to improvements in donor hemodynamics, allowing significant reduction in vasopressor support.

## Interventions

Participants were randomly assigned to receive high-dose levothyroxine, high-dose methylprednisolone, a combination of both, or no hormonal therapy (control).

## Participants

199 Consecutive adult organ donors.

## Outcomes

The primary outcome was the difference in vasopressor requirement to maintain goal hemodynamics among the four treatment groups. Secondary mechanistic outcomes included the assessment of thyroid hormone (TH) levels, cortisol levels and markers of inflammation (C-reactive protein [CRP] and multiple cytokines). Secondary clinical outcomes were the number, types, and proportion of organs procured versus consented, rate of transplantation of procured organs, and patient and graft outcomes of organ recipients exposed to the various treatments.

## Follow-Up

120 days.

## Jadad Score

3.

## Data Analysis

Available case analysis.

## Allocation Concealment

No.

## Trial Registration

ClinicalTrials.gov—NCT04528797.

## Funding Source

Non-industry funded.

## Clinical Impact Summary

The haemodynamic instability seen in many brain dead (DBD) donors is thought in part to result from disruption in the hypothalamo-pituitary axis, resulting in reduced levels of thyroid hormone and vasopressin [1]. For this reason, donor management often includes supplementation of thyroid hormones and vasopressin, and use of corticosteroids. Existing evidence as to the benefits of hormone replacement in the DBD donor is conflicting, with potential benefits of thyroid hormone and desmopressin administration seen in observational registry studies not borne out in prospective randomised controlled trials [2, 3].

In a recent issue of Transplantation, Van Bakel et al. report the results of a prospective randomised controlled trial of donor management in 199 brain-dead organ donors [4]. Donors were randomised to four groups: high-dose levothyroxine, high-dose methylprednisolone, combination therapy and no hormonal therapy. Vasopressor requirements were assessed using a validated score (the vasoactive-inotropic score; VIS). The reduction in VIS from baseline was significant in the methylprednisolone and combination groups, but no improvement was seen in the levothyroxine alone or control groups.

Unlike many donor intervention studies, the investigators were careful to report organ utilisation and graft outcomes for all groups. No differences were found between groups, although the study was not powered for these outcomes.

Of note, the study was not blinded and this may have contributed to significant crossover from other arms to the combination arm and possibly impacted inotrope use. However, the findings above were confirmed in both intent-to-treat and per-protocol analyses.

Overall, these results support the existing RCT evidence that thyroid hormone replacement alone does not improve cardiovascular stability in DBD donors, and that the largest impact on stability comes from corticosteroid use.
